# Supplementary material for: Image-Based Modeling of Blood Flow and Oxygen Transfer in Feto-Placental Capillaries
Source: PLoS One. 2016 Oct 27;11(10):e0165369. doi: 10.1371/journal.pone.0165369 (PMC5082864; doi:10.1371/journal.pone.0165369)
Supplement: S1 Appendix — A description of the methods used in the image analysis and 3D simulations to increase the reproducibility of the results. (PDF) [file pone.0165369.s002.pdf]

S1 Appendix

Conversion of surfaces to finite-element meshes

Three-dimensional representations of villous and capillary surfaces, reconstructed from confocal laser scanning microscopy (CLSM) images, were obtained in previous studies; the methods for image acquisition and reconstruction prior to the present study are described in detail in [1, 2] and in the main section of the paper. The images were provided as either Virtual Reality Modeling Language (VRML) files in the format ".wrl", or in Polygon File Format as ".ply" files.

The surfaces were imported into *Meshlab* (version 1.3.3, http://meshlab.sourceforge.net), an open-source software package for editing 3D meshes, in which they were processed and output as ".stl" files. The processing applied in *Meshlab* was specific to each surface, with some requiring smoothing via the "Surface Reconstruction: Poisson" algorithm. For this algorithm, the default settings were edited to ensure that the surfaces were not oversmoothed, leading to a loss of detail; typically, "Octree Depth" was set to 12 and "Samples per Node" was set to 0.1. In some cases, smoothing led to joining of closely adjacent capillary surfaces, which was rectified by deleting the triangular faces joining the two capillaries and applying the "Close Holes" algorithm to fill in the resulting gaps in the surfaces. Some of the surfaces were too large or complex to be directly imported into *Comsol*; these surfaces were imported into the software package *Netfabb* (Basic version 6.4.0, http://netfabb.com), where unnecessary parts were cut away by a planar cutting algorithm, before processing in *Meshlab*.

Processed surfaces were imported into *Comsol Multiphysics* (version 5.2, http://comsol.com) as meshes via the "Mesh" node, with separate meshes created for the capillary and villous surfaces. The "Create Geometry from Mesh" option was selected to create 3D geometries from the meshes. The "Simplify mesh" option was selected to ensure the geometry was not split into a large amount of separate boundaries. The villous surface was converted to a villous volume by adding "Convert to Solid" to the "Geometry" node. To provide a flat surface where boundary conditions could be applied, the geometry was partitioned by a plane. To do this, a "Work Plane" was created in the "Geometry" node with the required position and orientation to minimize the effect on the geometries. Then a "Partition Objects" node was created to partition the solid object with the work plane. Finally, a "Delete Entities" node was created to delete the section of the geometry that was not required for computations.

The domain was meshed with tetrahedral elements. Separate meshes were created for solving for flow and oxygen transfer; the boundary layers expected to form in the oxygen transfer problem required a finer mesh to resolve, which was achieved by applying extra mesh refinement to the entire volume, especially at the capillary surface. The flow and oxygen transfer meshes typically consisted of $\sim 10^6$ and $\sim 10^7$ elements, respectively.

Simulations of blood flow and oxygen transfer

The simulations were performed by solving for blood flow for a specific pressure difference, followed by solving for oxygen transfer for a range of pressure differences. Blood flow in the capillary was modeled using the Stokes equations and boundary conditions as described in the main paper; outside the capillary, the velocity field was set to zero. When imposing the pressure at the inflow and outflow boundaries, the flow direction was specified to be normal to the boundary with the tangential velocity vector set to zero, as is common in flow simulations [3]. Oxygen transfer was modeled using the advection-diffusion equation with enhanced advection and boundary conditions as described in the main paper. The velocity field was specified to be equal to the solution of the Stokes equations previously calculated, multiplied by a factor depending on the pressure difference; as Stokes flow is linear, doubling the pressure difference simply doubles the magnitude of the solution without affecting the streamlines. The flow field was also multiplied by the enhancement to advection, which depended on the concentration when the nonlinear oxygen–hemoglobin dissociation law was taken into account. For both flow and oxygen transfer simulations, the solver was specified to be a "MUMPS" solver [4, 5], to avoid problems with multigrid solvers caused by the complicated 3D geometries.

As a consequence of this choice of solver, solutions had to be computed on a server node with a large amount of RAM (as described in the main paper). The advective flux through the outflow boundary or boundaries was calculated by including a "Surface Integration" under the "Derived Values" node and entering the normal velocity multiplied by the concentration (including oxygen bound to hemoglobin). Conservation of mass was checked by calculating the diffusive flux through the villous surface and confirming it to be equal to the advective flux through the outflow boundaries plus the diffusive flux through the inflow boundary. Diffusive fluxes were accurately calculated using the inbuilt "reacf" function in *Comsol*, which performed a summation of the corresponding components of the boundary residual vector over every node on the surface [6].

Capillary skeletonization

Capillary surface meshes were converted to tetrahedral meshes in the software package *Gmsh* (version 2.12, [7]). The ".stl" version of each capillary mesh was imported into *Gmsh* and a "Volume" was added by selecting "Elementary Entities" under the "Geometry" node. The tetrahedral meshes were exported in Nastran Bulk Data File (".bdf") format. The tetrahedral capillary meshes were imported into the image analysis software *Avizo* (Lite version 9.0, https://www.fei.com/software/avizo3d/). A regularized "Element Thickness" was generated by selecting "Volume rendering" of the ".bdf" file. An "Auto Skeleton" node was added to the regularized "Element Thickness", creating a "Spatial graph" of the skeletonized capillary. To generate a graph of the radius of the capillary at each point along the skeletonization line, a "Spatial graph statistics" node was added the the "Spatial graph".

Calculation of villous distance

The average distance of the capillary surface from the villous surface was calculated by importing a tetrahedral mesh of the villous volume in ".bdf" format into *Avizo*. A "Distance Map" node was created which, for each voxel in the villous volume, calculated the distance to the nearest villous surface voxel. The corresponding capillary surface was imported into *Avizo* using the corresponding ".stl" file. A "Surface Scalar Field" node was added to the capillary surface, with the input being the "Distance Map" previously created. The average distance of the capillary surface from the villous surface was calculated by creating a histogram of the "Surface Scalar Field" and taking the mean value; the standard deviation of the average villous distance was also calculated.

References

1. Jirkovská M, Kubínová L, Janáček J, Moravcová M, Krejčí V, Karen P. Topological properties and spatial organization of villous capillaries in normal and diabetic placentas. J Vasc Res. 2002;39(3):268–278. doi:10.1159/000063692.

2. Jirkovská M, Janáček J, Kaláb J, Kubínová L. Three-dimensional arrangement of the capillary bed and its relationship to microrheology in the terminal villi of normal term placenta. Placenta. 2008;29(10):892–897. doi:10.1016/j.placenta.2008.07.004.

3. Rannacher R. Finite Element Methods for the Incompressible Navier-Stokes Equations. In: Fundamental Directions in Mathematical Fluid Mechanics. eds. Galdi, GP, Heywood, JG and Rannacher, RB. Basel: Birkhäuser Basel.; 2000.

4. Amestoy PR, Duff IS, L'Excellent JY, Koster J. A fully asynchronous multifrontal solver using distributed dynamic scheduling. SIAM J Matrix Anal A. 2001;23:15–41. doi:10.1137/S0895479899358194.

5. Amestoy PR, Guermouche A, L'Excellent JY, Pralet S. Hybrid scheduling for the parallel solution of linear systems. Parallel Comput. 2006;32(1):136–156. doi:10.1016/j.parco.2005.07.004.

6. Heinrich JC, Pepper DW. Intermediate Finite Element Method: Fluid Flow and Heat Transfer Applications. Philadelphia, PA: Taylor & Francis; 1999.

7. Geuzaine C, Remacle JF. Gmsh: A 3-D finite element mesh generator with built-in pre-and post-processing facilities. Int J Numer Meth Eng. 2009;79(11):1309–1331.
