# Supplementary material for: Image-Based Modeling of Blood Flow and Oxygen Transfer in Feto-Placental Capillaries
Source: PLoS One. 2016 Oct 27;11(10):e0165369. doi: 10.1371/journal.pone.0165369 (PMC5082864; doi:10.1371/journal.pone.0165369)
Supplement: S2 Appendix — The derivation of the parameter B quantifying the enhancement to oxygen advection due to its affinity for hemoglobin. (PDF) [file pone.0165369.s003.pdf]

S2 Appendix

Estimation of the oxygen advection enhancement parameter B

In this Appendix the values of the parameters used in the derivation of the oxygen advection enhancement parameter $B = 1 + c_{\max} K k_{\text{hn}} / \rho_{\text{bl}}$ are estimated. The results are summarized in Table S2.1.

The value of $c_{\max}$ can be calculated by multiplying three quantities. The first quantity is the concentration of hemoglobin in the fetal blood $c_{\text{Hb}} \approx 0.155$ g/ml, corresponding to a fractional hematocrit of approximately 0.48 [1]. The second quantity is the fetal hemoglobin binding capacity β, also known as Hüfner's constant. Here it is assumed that $\beta \approx 1.42$ ml O_2/g Hb, giving the oxygen capacity of fetal blood to be $c_{\text{Hb}} \cdot \beta \approx 0.22$ ml O_2/ml blood, which is the value given for fetal oxygen capacity in [2]. The final quantity is the amount of oxygen, which at standard temperature and pressure is given by $k \approx 1/22.4$ mol/l. Thus

$$c_{\max} = c_{\text{Hb}} \cdot \beta \cdot k \approx 9.82 \text{ mol/m}^3.$$

The partial pressure of oxygen P_{O_2} in the plasma can be related to the concentration of oxygen c dissolved in the plasma by Henry's law

$$P_{O_2} = \frac{k_{\text{hn}}}{\rho_{\text{bl}}} c.$$

Here $\rho_{\text{bl}} \approx 1000$ kg/m^3 is the density of blood [3]. The coefficient k_{hn} can be estimated by considering the concentration of dissolved oxygen at a specific partial pressure of oxygen [4]; solubility of oxygen in fetal blood can be assumed to be the same as in maternal blood [1]. At a partial pressure of oxygen of $P_{O_2} = 13$ kPa, the concentration of dissolved oxygen is $c \approx 0.13$ mol/m^3 [5], giving $k_{\text{hn}} \approx 7.5 \times 10^5$ mmHg kg/mol.

The partial pressure of oxygen in the intervillous space (maternal side) is approximately 60 mmHg [6]; this is the maximum partial pressure of oxygen that the fetal blood is expected to be exposed to and provides the upper bound for the range of values over which the oxygen-hemoglobin dissociation curve is linearized to give $K = 0.019$ mmMg (see Fig 1D in the main text).

Combining the above parameters gives $B = 1 + c_{\max} K k_{\text{hn}} / \rho_{\text{bl}} \approx 141$, showing that fetal blood provides a larger enhancement to advection of oxygen than maternal blood, for which $B \approx 94$ [4].

Table S2.1. Parameters used in the derivation of the oxygen advection enhancement parameter B.

Parameter	Value	Reference
Diffusion coefficient of RBC (D_{RBC})	10^{-13} m^2/s	[7]
Concentration of hemoglobin in fetal blood (c_{Hb})	0.155 g Hb/ml blood	[1]
Fetal hemoglobin binding capacity (β)	1.42 ml O_2/g Hb	[2]
Amount of oxygen in moles per litre of oxygen (k)	1/22.4 mol/l	
Oxygen content of fetal blood at full saturation ($c_{\max} = c_{\text{Hb}} \cdot \beta \cdot k$)	9.82 mol/m^3	
Density of blood (ρ_{bl})	1000 kg/m^3	[3]
Henry's law coefficient (k_{hn})	7.5×10^5 mmHg kg/mol	[5]
Partial pressure of oxygen in the maternal blood in the intervillous space	60 mmHg	[6]
Gradient of linearized fetal oxygen-hemoglobin dissociation curve (K)	0.019 mmHg^{-1}	
Oxygen advection enhancement parameter $B = 1 + c_{\max} K k_{\text{hn}} / \rho_{\text{bl}}$	141	

References

1. Hill EP, Power GG, Longo L. A mathematical model of carbon dioxide transfer in the placenta and its interaction with oxygen. Am J Physiol. 1973;224(2):283–299.

2. Longo LD, Bartels H. Respiratory gas exchange and blood flow in the placenta. Bethesda, MD: Nat. Inst. Child Health Hum. Dev.; 1973.

3. Wang SH, Lee LP, Lee JS. A linear relation between the compressibility and density of blood. J Acoustic Soc Am. 2001;109(1):390–396. doi:10.1121/1.1333419.

4. Serov AS, Salafia CM, Brownbill P, Grebenkov DS, Filoche M. Optimal villi density for maximal oxygen uptake in the human placenta. J Theor Biol. 2015;364:383–396. doi:10.1016/j.jtbi.2014.09.022.

5. Law R, Bukwirwa H. The physiology of oxygen delivery. Update Anaesthesia. 1999;10(1-2):39.

6. Rodesch F, Simon P, Donner C, Jauniaux E. Oxygen measurements in endometrial and trophoblastic tissues during early pregnancy. Obstet Gyneco. 1992;80(2):283–285.

7. Higgins JM, Eddington DT, Bhatia SN, Mahadevan L. Statistical dynamics of flowing red blood cells by morphological image processing. PLoS Comput Biol. 2009;5(2):e1000288. doi:10.1371/journal.pcbi.1000288.
