# Supplementary material for: Image-Based Modeling of Blood Flow and Oxygen Transfer in Feto-Placental Capillaries
Source: PLoS One. 2016 Oct 27;11(10):e0165369. doi: 10.1371/journal.pone.0165369 (PMC5082864; doi:10.1371/journal.pone.0165369)
Supplement: S3 Appendix — Further numerical results generated using the idealized axisymmetric model, to quantify the effect of oxygen transfer through the surrounding villous volume upstream and downstream of the localized dilation. (PDF) [file pone.0165369.s004.pdf]

S3 Appendix

Effect of oxygen transfer upstream and downstream of a localized dilation

The effect of oxygen transfer upstream and downstream of the dilated section of the axisymmetric capillary is tested by relaxing the assumption of zero diffusive flux along the undilated sections γ_u of the capillary wall (see Fig 1E in the main paper). The boundary condition (8b) in the main paper is modified to

$$-D\frac{\mathrm{d}c}{\mathrm{d}r} = m(c - c_{\mathrm{mat}}) \quad \text{on } \gamma_u. \tag{1}$$

Here the oxygen transfer coefficient $m = N/Ac_{\mathrm{mat}}$ has been introduced, where A is the surface area of the capillary wall. The oxygen transfer on γ_u can be estimated by comparing m to the ratio between the diffusion coefficient D and the thickness d of the trophoblast layer separating the maternal and fetal blood. For $m \gg D/d$, the fetal blood is close to the maternal blood and the oxygen concentration is effectively fixed to be equal to the maternal oxygen concentration on the undilated sections of the capillary wall. For $m \ll D/d$, the trophoblast layer is so thick that the oxygen flux is effectively zero on the undilated sections of the capillary wall; setting $m = 0$ corresponds to the results with $c = 0$ on γ_u discussed in the main paper. Noting that $N \sim 10^{-6}\mu$g/s from Fig 2A in the main paper, taking the average of A and d from Table 3 in the main paper, and using the values of D and c_{mat} from Table 2 in the main paper, it is estimated that, on average throughout the fetal capillaries in this study, $m \approx 1.5D/d$. Increasing the value of m from $m = 0$ to $m = 2D/d$ is found to increase the optimal maximum radius of the localized dilation by around 10%, and the enhancement to oxygen transfer by less than 1% (Fig S3.1).

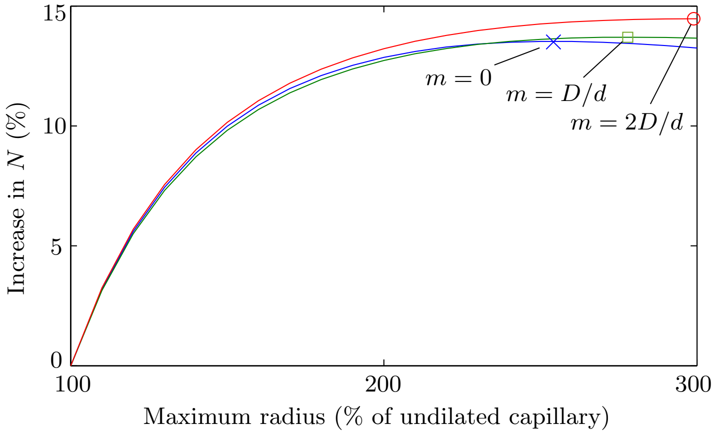

Fig S3.1. Increase in oxygen transfer rate N for a capillary dilation compared to a straight capillary, for a localized dilation with one degree of freedom, where the undilated radius is defined as R_{av} (see Fig 4A in the main paper). Oxygen transfer upstream and downstream of the dilation is taken into account via the oxygen transfer coefficient m. The optimal maximum radius is shown for $m = 0$ (blue cross), $m = D/d$ (green square) and $m = 2D/d$ (red circle), where D is the diffusion coefficient and d is the distance between the capillary surface and the villous surface, which is assumed to be constant along the undilated sections of the capillary.
